# Schwannoma Within a Muscular Nerve Branch to the Medial Head of Triceps: A Case Report

**DOI:** 10.1155/cro/9022134

**Published:** 2026-05-26

**Authors:** Jacobus Potgieter, Megan O′Connor, Leon Rajah

**Affiliations:** ^1^ Orthopaedics, Busamed Hillcrest Private Hospital, Durban, KwaZulu-Natal, South Africa; ^2^ Orthopaedics, Harry Gwala Regional Hospital, University of KwaZulu-Natal, Durban, KwaZulu-Natal, South Africa, ukzn.ac.za

**Keywords:** peripheral nerve sheath tumour, schwannoma, terminal branches, ulnar nerve branches in the arm, upper limb

## Abstract

**Introduction and Importance:**

Schwannomas are rare, benign, encapsulated tumours arising within peripheral nerve sheath Schwann cells. Only 19% of schwannomas occur in the upper limb, typically affecting major peripheral nerves on the flexor aspect distal to the elbow. No reports have documented a schwannoma originating from a muscular branch to the medial head of the triceps brachii. This case highlights a unique anatomical presentation and the diagnostic challenges associated with atypical schwannomas.

**Presentation of Case:**

A 37‐year‐old female presented with a 4‐year history of nonspecific neurological symptoms, including dysesthesia and swelling over the posteromedial aspect of her left arm. Initial investigations were inconclusive, but magnetic resonance imaging identified a tumour within the medial head of the triceps. Surgical excision revealed a well‐encapsulated tumour within a muscular nerve branch to the medial head of the triceps. Histopathology confirmed a schwannoma. Postoperatively, the patient experienced complete symptom resolution with no complications at 3 months.

**Clinical Discussion:**

The unusual location of the schwannoma may have contributed to the diagnostic delay. Cadaveric studies suggest that innervation to the medial head of the triceps can have several anatomic variations, including innervation from the ulnar nerve, the nerve in which schwannomas are most commonly located.

**Conclusion:**

This case underscores the importance of considering peripheral nerve tumours in patients with persistent, unexplained upper limb symptoms. Awareness of atypical anatomical variants and careful imaging interpretation are essential for timely diagnosis and effective management.

**Highlights**

• The schwannoma identified occurred in a muscular branch to the medial head of triceps—an unreported site.
• There was a 5‐year delay to management due to nonspecific symptoms and inconclusive imaging.
• The authors discuss that the clinical findings suggest that this schwannoma may have occurred in an anatomical variant of a muscular nerve to the triceps.
• Surgical excision led to complete symptom resolution.

## 1. Introduction

Schwannomas are rare, benign, encapsulated tumours originating from Schwann cells along peripheral nerve sheaths [1–9]. They are usually sporadic but can be heritable as part of genetic syndromes such as neurofibromatosis or schwannomatosis [10]. Schwannomas comprise only 5% of soft tissue tumours [2–7] but account for 90% of nerve tumours [2]. Anatomically, approximately 19% of all schwannomas are found in the upper limb [2, 4], predominantly affecting major peripheral mixed nerves [2–7]. Most frequently schwannomas are reported as occurring on the flexor aspects of the upper limb [2, 4], which Tang et al. propose is due to the associated higher concentration of nerve fibres in this location [4]. Additionally, the majority of schwannomas appear to occur distal to the elbow, with one investigation of upper limb schwannomas identifying 75% of lesions distal to the elbow [4].

There is often a delay to diagnosis which previous investigators have suggested is due to the rarity of the condition [1, 2], its slow growth [2] and indistinct clinical features [2, 4]. A palpable mass or swelling is often the first symptom, with or without associated pain and paraesthesia, and motor weakness occurs in a limited number of cases [1, 4]. Clinical examination commonly yields a nonspecific positive Tinel′s sign [2, 4], and less commonly the clinician may be able to delineate a mass that Tang et al. describe as ‘mobile in the longitudinal plane along the nerve route’ [4]. Although magnetic resonance imaging (MRI) is the preferred diagnostic imaging modality, for schwannomas of the upper limb MRI is only 85% sensitive and 50% specific [5], and not always available to the clinician [5]. The utility of MRI is in its ability to localise the origin of the lesion and adjacent anatomy for surgical planning [1–3, 5]. The more readily available ultrasonography (US) can be used in the absence of MRI, to localise a lesion and determine its relationship to adjacent structures [2, 3, 5]. US efficacy for diagnosis is also limited by poor specificity and is operator dependent [3, 5]. Preoperative incisional biopsy in peripheral nerve tumours remains controversial due to the potential complications [3, 6, 11]. However, El Sayed et al. report employing it selectively, to rule out sarcoma in cases with inconclusive MRI findings [6].

Regarding management, investigations report excisional biopsy under magnification (loupe or microscope), through meticulous microsurgical enucleation, or with fascicular or nerve resection when nerve preservation is not possible, for symptomatic relief [1, 3, 6]. With this management, patients generally have improved symptomatology, low recurrence rates and limited complications [1, 6].

This investigation aims to report an upper limb schwannoma case distinguished by its unusual site of origin (the authors are unaware of a published report of a schwannoma originating from a muscular branch to the medial head of the triceps brachii) and diagnostic challenge, with the intention of expanding awareness of atypical schwannoma presentations and guiding clinicians in evaluating persistent, unexplained upper limb symptoms. This case report has been reported in line with the SCARE Criteria [12].

## 2. Case Presentation

A 37‐year‐old female with no prior significant medical history initially presented to an orthopaedic spine surgeon with dysesthesia of the left arm, forearm and hand for approximately 4 years. After clinical examination excluded cervical spine origin of the dysesthesia, the patient was referred to a neurologist for further assessment.

The neurologist performed a neurological examination of the upper limb and found no objective motor weakness; however, the patient subjectively reported dysesthesia of the arm. The dysesthesia was associated with a subtle swelling over the posteromedial aspect of the arm distally and radiated caudally over the medial epicondyle towards the ulnar aspect of her forearm and hand. A comprehensive set of special investigations were conducted to determine the origin of the swelling and dysesthesia. Plain radiographs of the humerus and elbow did not reveal an identifiable pathology. Nerve conduction studies identified no electrophysiological evidence of nerve compression or conduction deficits of the left radial, ulnar and median nerves. MRI of the cervical spine was conducted to confirm the absence of cervical pathology. Although the MRI revealed broad‐based central disc protrusions at levels C5‐C6 and C6‐C7, respectively, these findings were deemed to be clinically insignificant and not contributory to the symptoms experienced by the patient (which would have been more in keeping with pathology at C7‐T1 and T1‐T2 levels).

**Figure 1:**
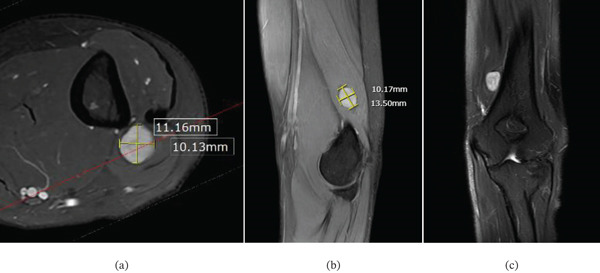
(a) T2 axial cut MRI image depicting the hyperintense tumour located within the medial head of the triceps brachii measuring 10 mm in the anteroposterior direction and 11 mm transversely. (b) A fat saturated sagittal MRI image depicting the hyperintense lesion measuring 13.5 mm craniocaudally. (c) A fat saturated coronal MRI image following contrast demonstrating tumour enhancement.

The MRI report of the left elbow documented the presence of a tumour located approximately 30 mm proximal to the triceps insertion. The images (Figure 1) showed the tumour measuring 10 mm (anteroposteriorly) by 11 mm (transversely) by 13.5 mm (craniocaudally). The report further detailed that the tumour appeared encapsulated and was located within the muscle belly of the medial head of triceps. The imaging lacked several hallmark features of schwannoma, including the absence of a target sign and the inability to identify a nerve entering or exiting the lesion. Although a split‐fat sign was present on sagittal T1 imaging, this finding is nonspecific and may be seen in other intramuscular tumours. Additionally, no anatomical relationship was demonstrated between the mass and the radial, ulnar or median nerves. The lesion was T1‐isointense, T2‐hyperintense, and exhibited diffuse, predominantly homogeneous enhancement without septations or a pseudocapsule. Taken together, these features favoured an initial diagnosis of intramuscular myxoma rather than schwannoma.

Following these investigations the patient was referred to an orthopaedic shoulder and elbow surgeon for assessment and management. The patient volunteered that she could recollect a prior trauma to the elbow, which preceded her symptoms by several years. She had no recent trauma history. The findings of the clinical examination and special investigations were discussed with the patient. Considering the mild clinical symptoms, slow growth, lack of electrophysiological evidence of nerve compression and benign appearance on imaging of the tumour, the patient was offered, and requested, symptomatic management. Following failure of symptom resolution after 1 year of this management, and with the concern that the tumour was enlarging, the patient returned to the surgeon for intervention. Excisional biopsy was discussed during consultation. The patient was agreeable to the procedure and surgical removal in theatre was planned. US was ordered preoperatively to confirm the location of the lesion and to plan surgical excision.

**Figure 2:**
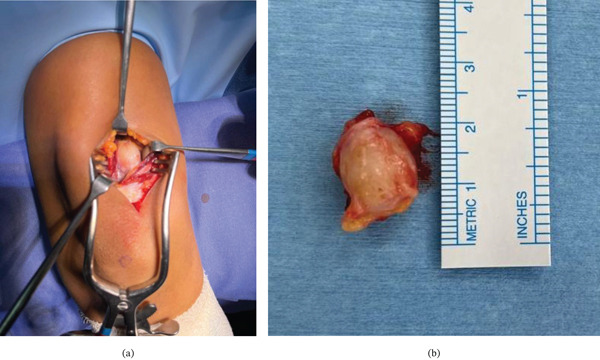
(a) Intraoperative photographs depicting the surgical approach and the excised lesion. The patient positioned prone with head proximally and the hand distally. The incision is on the ulnar aspect of the arm, directly overlying the lesion. (b) The encapsulated lesion measuring approximately 16 mm in length.

Surgery was performed under general anaesthesia. The patient was positioned prone, and a tourniquet was applied. A vertical incision was made on the ulnar aspect of the arm overlying the distal portion of the medial head of triceps brachii (where the tumour was identified with US). The skin and fascia were incised and then intramuscular blunt dissection was performed within the medial head of triceps, approximately 30 mm from the insertion, towards the tumour location. The tumour was identified arising within a nerve branch to the medial head of the triceps brachii (Figure 2). A nerve stimulator was not available intraoperatively, as the nerve′s association with the tumour had not been anticipated preoperatively. A superficial epineurial incision was performed to access the tumour which had a secondary tumour capsule, as such extracapsular enucleation was possible with no fascicles adherent to the tumour capsule. Loupe magnification was utilised for the dissection, and the surgeon was able to confirm fascicular continuity post excision.

**Figure 3:**
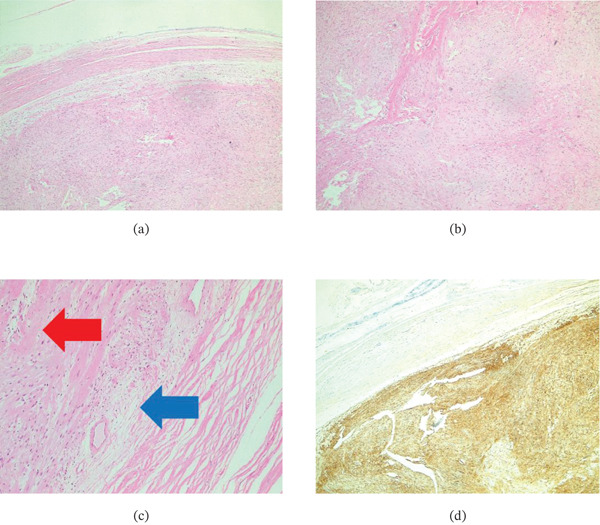
(a) Histological findings of the excision biopsy. Enucleation confirmed as the entire specimen found to exhibit a continuous capsule. (b,c) The tumours′ hypercellular and hypocellular areas, respectively, that consist of sheets and whorls of spindle cells. Myxoid (blue arrow) change and areas of hyalinised fibroses (red arrow) are evident in hypocellular areas. No necrosis or degenerate atypia was observed. (d) Diffuse expression of the S100 stain in keeping with a diagnosis of schwannoma.

The tumour was sent for histological examination (Figure 3). The schwannoma was finally diagnosed on histopathological assessment. Histopathology showed well‐organised hypercellular Antoni A areas with Verocay bodies and hypocellular Antoni B areas characteristic of schwannoma. The tumour was encapsulated, showed strong diffuse S‐100 positivity, and demonstrated no mitotic activity (0/10 high‐power fields), supporting exclusion of neurofibroma.

Immediately postoperatively the patient reported complete resolution of the dysesthesia with no motor deficit. She was discharged home and had an unremarkable postoperative course. At the 6 weeks postoperative review, the surgical scar had healed with no sensitivity, and the resolution of her symptoms was maintained. At the final follow‐up 3 months postsurgery, no further imaging was planned, the patient was discharged, and the authors are unaware of any subsequent complications.

## 3. Discussion

We present a rare case of a symptomatic schwannoma located in a muscular nerve branch to the medial head of triceps brachii. Although previous investigations have documented schwannomas located in the terminal branches of major peripheral nerves, such as Tang et al., who reported two schwannomas located within the dorsal cutaneous branch of the ulnar nerve and the superficial branch of the radial nerve, respectively [4], at the time of writing, the authors are unaware of a previous investigation identifying a schwannoma at the same location as documented within this report. Furthermore, several investigations highlight that schwannomas affecting the radial nerve are particularly rare [2, 8, 9, 11, 13].

This assumes that the schwannoma was located within a terminal branch of the radial nerve to the medial head of the triceps brachii. Based on several anatomical investigations [14–16], the distribution of the dysesthesia on the ulnar aspect of the patient′s forearm and hand, and the location of the lesion approximately 30 mm from the triceps insertion [14, 16], other anatomical possibilities considered include an aberrant ulnar contribution; however, definitive nerve origin could not be established intraoperatively. Cadaveric studies have demonstrated that in some cases the medial head of the triceps brachii receives partial innervation from the ulnar nerve [14–16]. Furthermore, the most distal branches from the radial nerve entering the medial head of triceps do so approximately 63.03 mm from the distal humerus intercondylar line [16], where the most distal branches of the ulnar nerve do so at 31 mm, approximately the location of the presently reported schwannoma [14]. Due to the additional dissection and wound size necessary to trace the nerve proximally, this was not performed. Given that the ulnar nerve is the most frequently reported site for schwannomas in the upper limb, this anatomical variation provides a plausible justification for the tumour′s origin in this case [2–5].

An additional point of interest to the authors was the patient′s admission of prior trauma to the affected area. An investigation by Jabaly et al. has brought into question the possibility that local tissue trauma may have a relationship to the development of a schwannoma, through the inflammatory Schwann cell overgrowth response mediated by cytokines or through mutations [17], but presently, no causal relationship has been established [17].

The atypical location likely contributed to the diagnostic challenge the case posed. The definitive diagnosis was made histologically, as no investigation identified schwannoma as a differential. This is congruent with previous investigation that refers to the diagnostic challenges of identifying schwannomas in uncommon locations [2]. One interesting finding of Istefan et al. that may be of use diagnostically was the finding of a strong correlation between the length and width of schwannomas in their MRI investigation [5]. Schwannomas were axially longer than they were transversely [5].

Regarding surgical excision of identified schwannomas, the literature supports excision for symptomatic relief, definitive histological diagnosis and for prevention of advancement of neurological deficit from growth [5, 6]. As in the case of the patient presented, improvement in dysesthesia and other neurological symptoms has been reported following excision [1, 6]. Recurrence rates have been uniformly low (0%–0.6%) in previous investigations [2, 6].

Possible limitations to this study include the small sample size of a single case due to this rare presentation. Comparisons had to be made to larger studies with more common presentations of the same pathology. Failure to identify a peripheral nerve tumour preoperatively could have impacted the outcome if a less experienced surgeon attempted removal.

## 4. Conclusion

This case report documents a rare schwannoma arising from a muscular nerve branch to the medial head of the triceps brachii, an anatomical site not previously described. The atypical location, coupled with nonspecific symptoms and inconclusive imaging, contributed to diagnostic delay. This case underscores the importance of considering peripheral nerve tumours in the differential diagnosis of persistent upper limb symptoms.

## Author Contributions

**Jacobus Potgieter:** data analysis, first draft preparation, manuscript revision. **Megan O′Connor:** study design, manuscript preparation and review. **Leon Rajah:** study conceptualisation, manuscript review.

## Funding

No funding was received for this manuscript.

## Disclosure

The authors declare authorship of this article and that they have followed sound scientific research practice. This research is original and does not transgress plagiarism policies.

## Ethics Statement

The author declares that this submission is in accordance with the principles laid down by the Responsible Research Publication Position Statements as developed at the 2nd World Conference on Research Integrity in Singapore, 2010, and was conducted in accordance with the Declaration of Helsinki (1964). The study complied with the South African Department of Health ethics guidelines (2015). Written informed consent was obtained from the patient to use the clinical history from the case record, radiographs, imaging reports and photographs taken intraoperatively with the purpose of publication. Formal institutional ethical approval was not required.

## Consent

All the patients allowed personal data processing and informed consent was obtained from all individual participants included in the study.

## Conflicts of Interest

The authors declare no conflicts of interest.

## Data Availability

The data that support the findings of this study are available on request from the corresponding author. The data are not publicly available due to privacy or ethical restrictions.
